# A Potential biomarker for the early diagnosis of OSCC: saliva and serum PrP^C^

**DOI:** 10.7150/jca.92489

**Published:** 2024-01-21

**Authors:** Jun Zheng, Kaixiong Chen, Lanyu Cai, Yangyang Pan, Yan Zeng

**Affiliations:** 1 Department of Stomatology, Central People's Hospital of Zhanjiang, Zhanjiang, 524037 Guangdong, China.; 2 Department of Otolaryngology, Central People's Hospital of Zhanjiang, Zhanjiang, 524037 Guangdong, China.; 3 Precision Clinical Laboratory, Central People's Hospital of Zhanjiang, Zhanjiang, 524037 Guangdong, China.

**Keywords:** Oral squamous cell carcinoma, PrP^C^, Saliva, Serum, Diagnosis

## Abstract

**Background:** Oral squamous cell carcinoma (OSCC) is frequently diagnosed at an advanced stage, and the high mortality of patients is mainly due to the delay of diagnosis. Cellular prion protein (PrP^C^) contributes to the occurrence and development of many malignant tumors. However, little has been known about the clinical and diagnostic value of PrP^C^ in OSCC. This study investigated the levels of PrP^C^ in the saliva and serum of patients with OSCC, OPMD and control group and their diagnostic value.

**Methods:** The Cancer Genome Atlas (TCGA), Gene Expression Omnibus (GEO) and Clinical Proteome Tumor Analysis Consortium (CPTAC) databases were analyzed to evaluate the expression of human prion protein gene (*PRNP*) mRNA and PrP^C^ in OSCC. Enzyme-linked Immunosorbent Assay (ELISA) was utilized to detect the expression of PrP^C^ in saliva and serum samples of OSCC, OPMD and control groups. Furthermore, diagnostic value and clinical significance of PrP^C^ in OSCC was identified. Protein-protein interaction (PPI) network was constructed by STRING. GO and KEGG analysis were performed by ClusterProfiler.

**Results:** The levels of *PRNP* mRNA and PrP^C^ in OSCC were significantly higher than those in the control group from databases (*P*<0.05). Besides, salivary and serum PrP^C^ of OSCC patients showed increased levels compared with OPMD and control groups (*P*<0.05). The expression of salivary and serum PrP^C^ of OSCC was correlated with the degree of differentiation (*P*<0.05), and the expression of PrP^C^ from CPTAC was related to tumor stage of OSCC (*P*<0.05). The areas under the diagnostic curves (AUCs) of salivary and serum PrP^C^ were 0.807 and 0.671, respectively. GO and KEGG analysis revealed that PrP^C^ might be related to cell adhesion, cell differentiation, signal transduction and apoptosis, and participate in the pathways of focal adhesion, PI3K-Akt signaling pathway and ECM- receptor interaction in OSCC.

**Conclusion:** PrP^C^ in saliva and serum may be a potential biomarker for early diagnosis of OSCC.

## Introduction

Oral squamous cell carcinoma (OSCC), which accounts for more than 40% of head and neck malignancies, ranks the sixth most common cancer worldwide 1. According to the latest report, 377,713 new OSCC cases and 177,757 deaths have been estimated worldwide in 2020 2. The 5-year survival rate following early detection is better than 90%, whereas that following late detection is only 30%, so the 5-year survival rate remaining less than 50% over the last 50 years 3. OSCC is frequently diagnosed at an advanced stage, and the high mortality of patients is mainly due to the delay of diagnosis 4, suggesting that early diagnosis of OSCC remains one of the most effective strategies to improve the treatment and prognosis of the disease. A large number of articles have demonstrated that serum may contain reliable biomarkers for detecting OSCC, such as Porphyromonas gingivalis IgG, interleukin 6, Cripto-1 and Endothelin-1 5-7. However, the usefulness of serum biomarkers is unsatisfactory due to their low diagnostic sensitivity and specificity in the early stage of OSCC. Therefore, a more effective, reliable and convenient diagnostic method is urgently needed in the early diagnosis of OSCC. Compared with serum samples, salivary samples play a more critical role in early diagnosis, because salivary samples are easy to store with little tendency to condense and can be safely handled. Meanwhile, saliva is collected locally from sites close to oral cancers, which enables the detection and analysis of tumor-specific biomolecules with minimal interference 8-10. In this context, saliva has been used as a potential tool for disease diagnosis, including pancreatic cancer, HIV, Sjögren's syndrome, Hepatitis A, B and C, Alzheimer disease and diabetes mellitus 11, and it has frequently attracted more and more attention as an important diagnostic fluid in oral cancer.

Cellular prion protein (PrP^C^), encoded by the human prion protein gene (*PRNP*), is a small glycoprotein that is anchored to the cell membrane outer leaflet via a glycosylphosphatidylinositol (GPI) tail 12, 13. Its pivotal role was originally identified in the pathogenesis of prion diseases 14. Additionally, it has recently been acknowledged to act on other neurodegenerative disorders as Parkinson's and Alzheimer's diseases 14. Interestingly, it has also contributed to the occurrence and development of malignant tumors such as gastric cancer 15, pancreatic cancer 16, breast cancer 17 and so on, as well as potentially supporting drug resistance in gastric cancer 18 and breast cancer 19. Our team has demonstrated that PrP^C^ is overexpressed in OSCC tissues by using immunohistochemical techniques, and known from the findings that it may be an instrumental biomarker for the diagnosis and treatment of patients with OSCC 20. This study is a continuation of our previous work to assess the expression and diagnostic significance of PrP^C^ in patients with OSCC. More importantly, the present study indicates the diagnostic value of PrP^C^ in saliva and serum of patients with OSCC and oral potentially malignant disorders (OPMDs) for the first time.

Through the Cancer Genome Atlas (TCGA), Gene Expression Omnibus (GEO) and Clinical Proteome Tumor Analysis Consortium (CPTAC) databases, this study explored the expression and diagnostic value of *PRNP* between OSCC and normal tissues from mRNA and protein levels. We further used Enzyme-linked Immunosorbent Assay (ELISA) to detect the levels of PrP^C^ in saliva and serum in OSCC, OPMD and control participants, and verified the diagnostic value and clinical significance of salivary and serum PrP^C^ in OSCC. Meanwhile, the potential functions and signaling pathways of PrP^C^ were subsequently revealed, which may explain the role of PrP^C^ in tumorigenesis and development, so as to provide evidence for further research that this protein is a potential biomarker and possible therapeutic target of OSCC.

## Materials and Methods

### Study Population

All patients signed an informed consent and the present project was approved by the Central People's Hospital of Zhanjiang (Zhanjiang, China). The study groups included 76 patients with OSCC (age range from 48-85), 30 patients with OPMDs (age range from 42-79), including 13 cases of lichen planus, 12 cases of leukoplakia and 5 cases of erythroplakia. 78 healthy individuals that are cancer-free and without OPMDs served as the control group (age range from 40-78). Patients were originally diagnosed by two pathologists from the Central People's Hospital of Zhanjiang. All samples were collected before operation. The characteristics of participants in each group were listed in Table 1.

### Saliva collection

Salivary samples were collected during 9:00 to 12:00 AM under a non-stimulatory condition, using previously described methods 21. Subjects were asked to rinse their mouth with water at least 5 minutes prior to saliva collection. Collected saliva into the sterile disposable tubes, then the samples were centrifuged at 1,000 × g for 2 minutes at room temperature (RT) and the supernatant was collected into 1.5 ml sterile sample tubes. All salivary samples were stored at -80℃.

### Serum collection

Fasting Blood samples of participants were obtained from 8:00 to 11:00AM, and centrifuged at 1200 × g for 10 minutes at RT and the supernatant was collected into 1.5 ml sterile sample tubes. All serum samples were stored at -80℃.

### Enzyme-linked Immunosorbent Assay (ELISA)

The content of PrP^C^ in saliva and serum samples was detected by ELISA. PrP^C^ WH2 antibody (Affiliated Cancer Hospital, Institute of Guangzhou Medical University) was added to the appropriate wells of ELISA plates, incubated overnight at 4 ℃ and blocked with 10% fetal bovine serum. After removal of the liquid from each well, 200 μl aliquot of each saliva or serum sample or 200 μl of PrP^C^ standard solution was added into each well for 2 hours, then coated with biotin labeled secondary antibody (Affiliated Cancer Hospital, Institute of Guangzhou Medical University). Next, washed and added streptavidin (solarbio, Beijing, China) into wells for 30 minutes at RT, then washed and added 100 μl of TMB substrate solution to each well. After that, the plates were kept at RT for 15 minutes. In the end, 100 μl stop solution was added to each well to terminate the enzyme reaction, and the absorbance was read at 450 nm by using a spectrophotometer.

### Data sources and extractions

Expression of *PRNP* mRNA in head and neck squamous cell carcinoma (HNSCC) was obtained through TCGA RNA-seqV2 data (The Cancer Genome Atlas Research Network; http://cancergenome.nih.gov), and GSE 30784 dataset of GEO (Gene Expression Omnibus; https://www.ncbi.nlm.nih.gov/geo/). Expression of PrP^C^ was sourced from CPTAC database (Office of Cancer Clinical Proteomics Research, https://cptac-data-portal.georgetown.edu/) 22, 23. The non-oral squamous cell carcinoma samples were excluded from these obtained HNSCC samples. Therefore, there were 331 OSCC samples and 32 samples adjacent to cancer from TCGA, 167 OSCC cases and 45 normal tissue cases from GSE 30784, 53 OSCC samples and 24 normal tissue samples from CPTAC. The clinical information was downloaded from TCGA and CPTAC for subsequent analysis. Differential expression of *PRNP* mRNA in various cancer tissues compared with control normal tissues was extracted via Gene Expression Profiling Interactive Analysis (GEPIA), an online tool based on TCGA and the Genotype-Tissue Expression (GTEx) (Gene Expression Profiling Interactive Analysis, http://gepia.cancer-pku.cn/detail.php?gene=PRNP) 24.

### PrP^C^-related proteins interaction network analysis

The STRING: functional protein association networks, was used to establish protein-protein interactions (STRING; http://string-db.org/, version 11.5) 25. Searching the term 'PrP^C^' or '*PRNP*' of homo sapiens from the full string network with the highest confidence (0.9), PrP^C^-related proteins were obtained.

### Differentially expressed proteins (DEPs) analysis

DEPs were regarded as differentially expressed PrP^C^-related proteins between OSCC and control groups from CPTAC database. Student's t test was performed by R software (version.4.1.1) to screen the DEPs.* P*<0.05 was considered as significant difference.

### Gene Ontology (GO) and Kyoto Encyclopedia of Genes and Genomes (KEGG) enrichment analysis

The functions and signaling pathways of DEPs in OSCC were explored by GO and KEGG enrichment analysis using ClusterProfiler, which is a R package for visualization of the results 26-28. By Fisher's exact test, corrected p value less than 0.05 was considered to have significant enrichment.

### Statistical analysis

All results were presented as the mean ± standard deviation (SD). Student's t test was used to compare the significant differences between any two groups. One-Way ANOVA was used to evaluate different levels of serum and salivary PrP^C^ among OSCC, OPMD and control groups. The Fisher's exact test was performed for GO and KEGG enrichment analysis. Through the construction of receiver operating characteristic (ROC) curves, the diagnostic value of PrP^C^ and *PRNP* mRNA were compared by calculating the areas under the ROC curves (AUCs). Reference cut-off values were estimated by Youden indexes. Differences were considered statistically significant when *P* < 0.05. All analyses were carried out with SPSS (version 20.0, Armonk, NY, USA), GraphPad Prism (version 8.0.1, Bethesda, MD, USA) and R software (version.4.1.1, R foundation, Vienna, Austria).

## Results

### *PRNP* mRNA expression in multiple types of cancers

**Figure 1:**
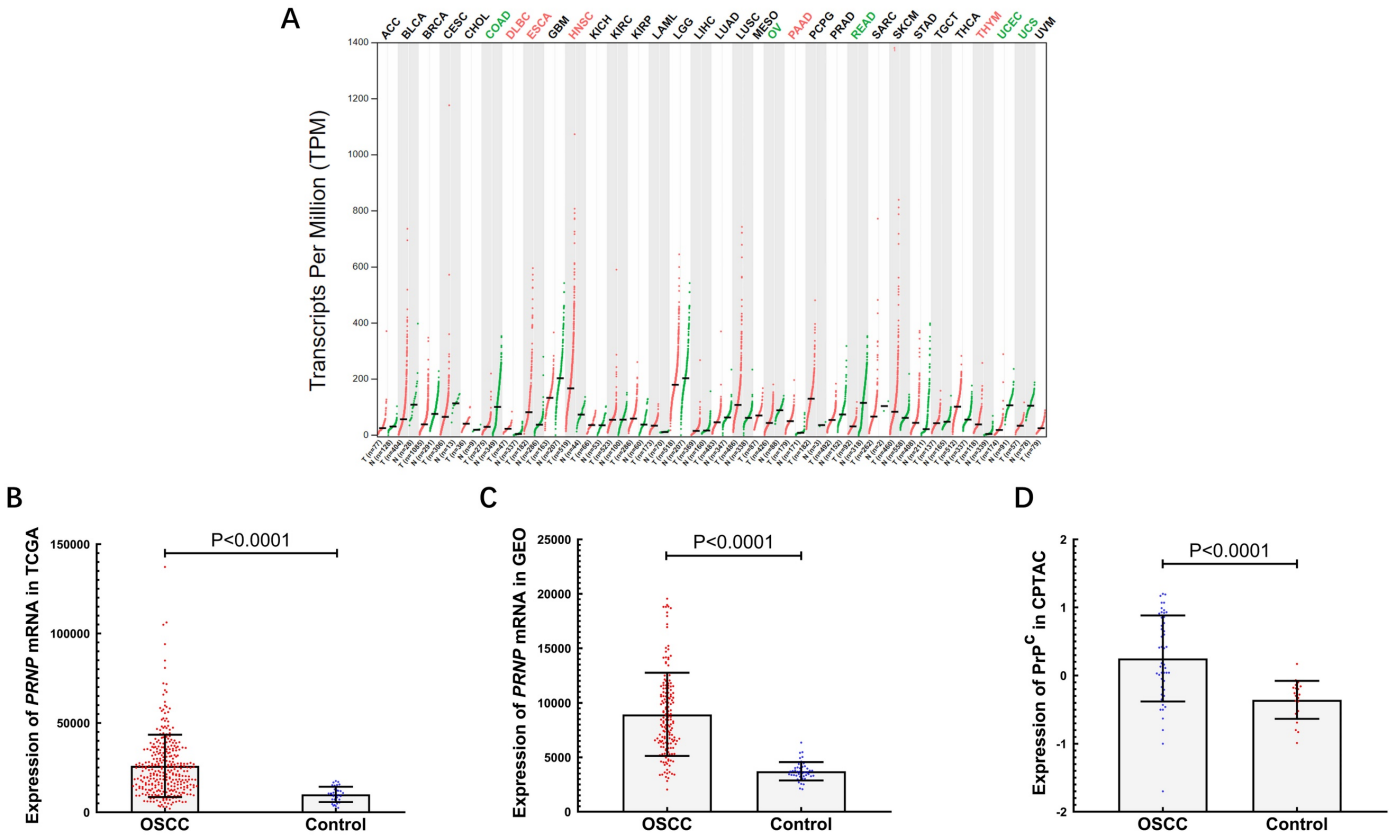
The expression of *PRNP* and PrP^C^ in databases. (A) *PRNP* expression profile across 33 types of tumor samples and paired normal tissues. Each dots represent expression of samples. Red font denotes up-regulation of *PRNP* in the cancers, and green font denotes down-regulation of *PRNP* in the cancers. (B) The expression of *PRNP* mRNA in OSCC and control groups from TCGA. (C) The expression of *PRNP* mRNA in OSCC and control groups from GEO. (D) The expression of PrP^C^ in OSCC and control groups from CPTAC.

The expression of *PRNP* mRNA in 33 kinds of cancer and their adjacent normal tissues was compared via GEPIA (Fig. 1A). The level of *PRNP* mRNA was down-regulated in five types of cancers (colon adenocarcinoma, ovarian serous cystadenocarcinoma, rectum adenocarcinoma, uterine corpus endometrial carcinoma and uterine carcinosarcoma), whereas its level was up-regulated in other fives (lymphoid neoplasm diffuse large B-cell lymphoma, esophageal cancer, head and neck squamous cell carcinoma, pancreatic adenocarcinoma and thymoma). Among various tumor tissues, the *PRNP* mRNA expression is the highest in low-grade glioma, followed by squamous cell carcinoma of head and neck, while its expression is the lowest in hepatocellular carcinoma.

### Expression of *PRNP* mRNA from TCGA and GEO, and expression of PrP^C^ from CPTAC

The level of *PRNP* mRNA in OSCC tissues is much higher than that in paracancerous or normal tissues from TCGA and GEO data (*P* < 0.0001) (Fig.1B-C). As the same, PrP^C^ level in OSCC is obviously higher than that in normal tissues from CPTAC (*P* < 0.0001) (Fig. 1D).

### The levels of salivary and serum PrP^C^ in OSCC, OPMD, and control groups

**Figure 2:**
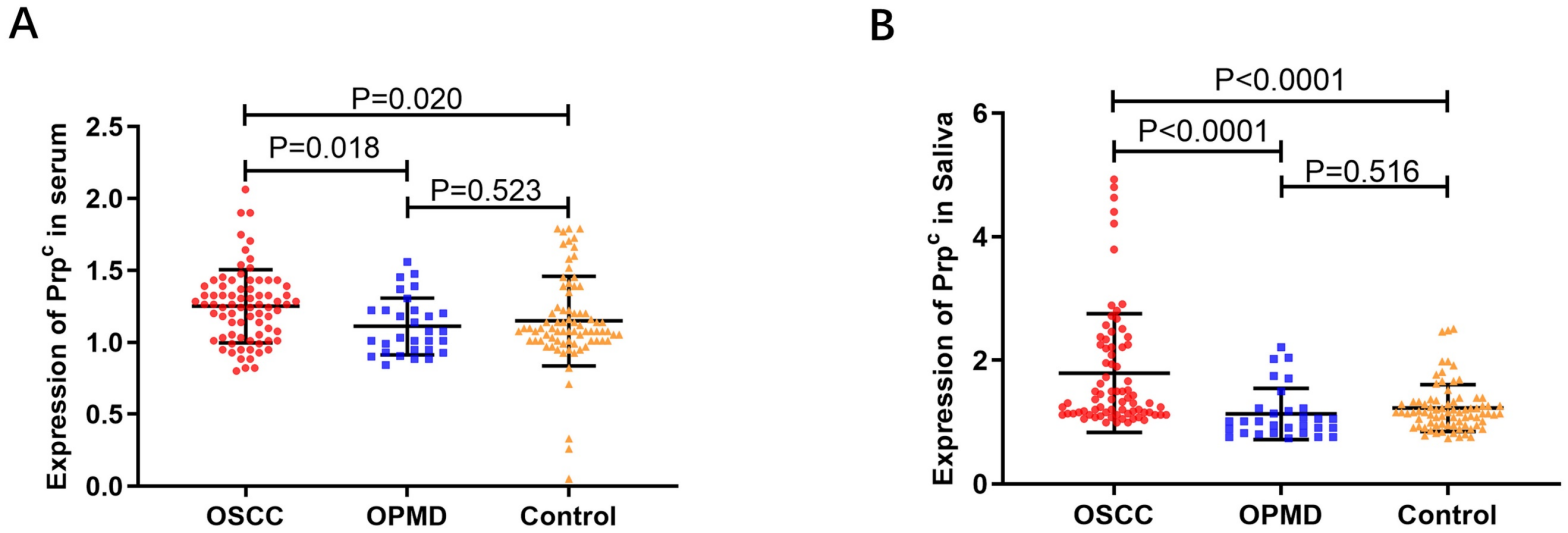
Scatter plots showing the expression of PrP^C^. (A) The expression of PrP^C^ in serum of OSCC, OPMD and control groups. (B) The expression of PrP^C^ in saliva of OSCC, OPMD and control groups.

As shown in Fig. 2A-B and Table 2, salivary and serum levels of PrP^C^ in OSCC patients were higher than those in the OPMD and control groups (*P* < 0.05), but there were no significant differences between OPMD patients and normal controls (*P* > 0.05).

### Association of *PRNP* mRNA and PrP^C^ levels with clinicopathological parameters

**Figure 3:**
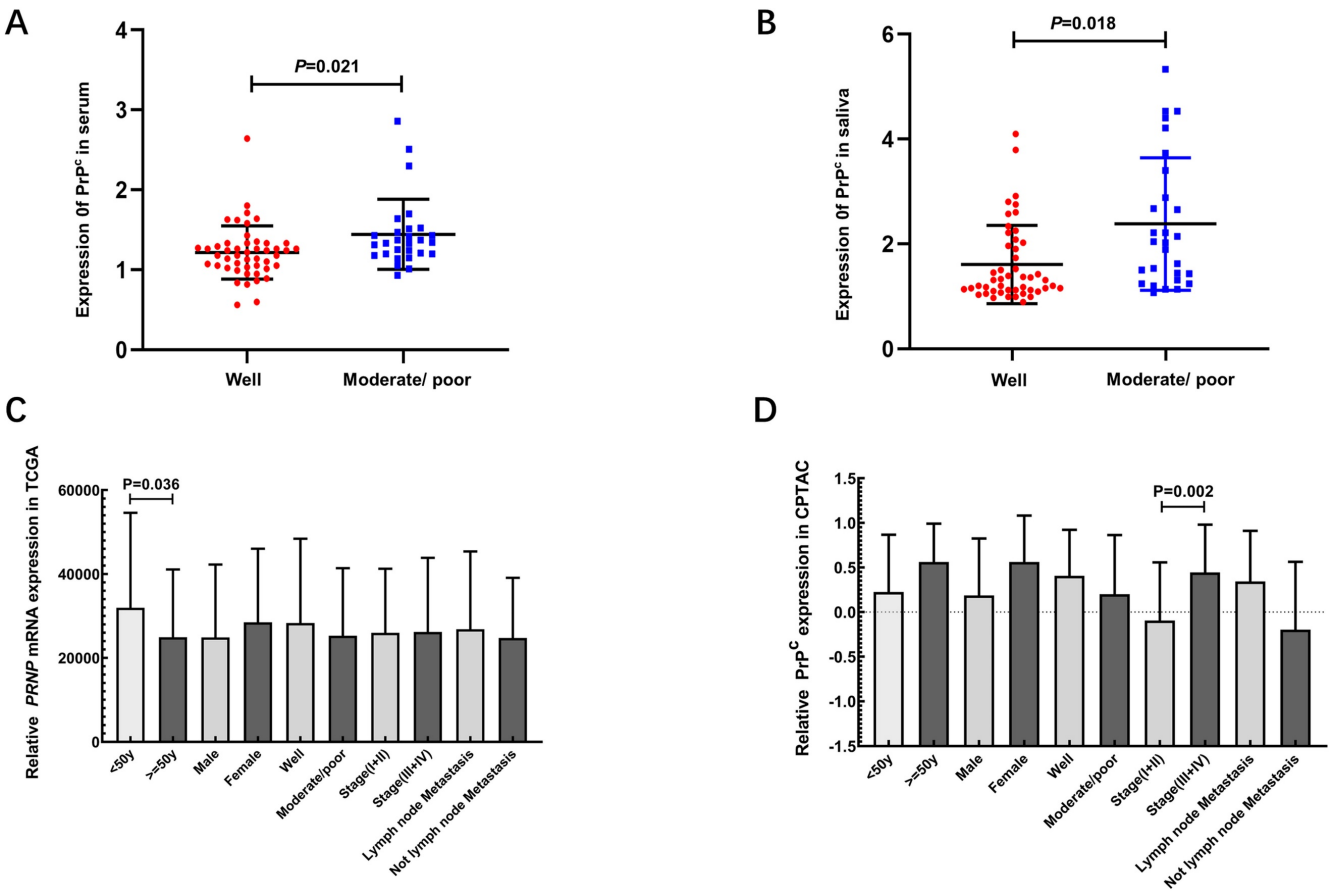
Associations between *PRNP* mRNA and PrP^C^ and clinicopathological variables of OSCC. (A) Scatter plots showing the expression of PrP^C^ in serum of OSCC patients with different degrees of differentiation. (B) The expression of PrP^C^ in saliva of OSCC patients with different degrees of differentiation. (C) Associations between *PRNP* mRNA and clinicopathological variables of OSCC in TCGA. (D) Associations between PrP^C^ and clinicopathological variables of OSCC in CPTAC.

The relationships between *PRNP* mRNA and PrP^C^ levels of OSCC patients and clinical indexes were as illustrated in Fig. 3 and Table 3. For salivary and serum samples, PrP^C^ levels showed significant correlation with differentiation (*P* < 0.05) (Table 3), and the expression of PrP^C^ in serum and saliva of OSCC patients with moderate or poor differentiation was higher than that of well differentiation (*P* < 0.05) (Fig. 3A-B). According to TCGA, *PRNP* mRNA expression was related to patients' age (*P* < 0.05) (Fig. 3C). In CPTAC, an obvious association was observed between PrP^C^ level and tumor stage (*P* < 0.01) (Fig. 3D).

### The diagnostic value of *PRNP* mRNA and PrP^C^ in databases between OSCC and normal tissues

**Figure 4:**
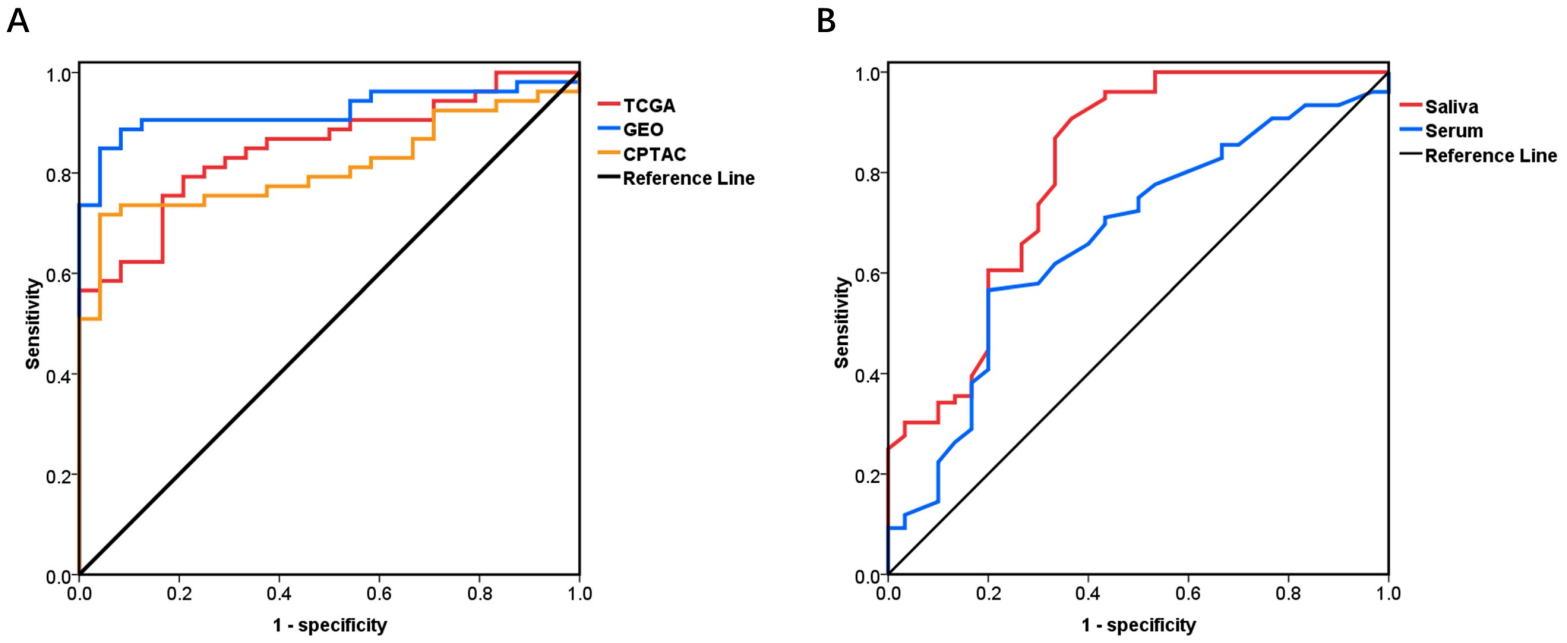
The diagnostic curves. (A) *PRNP* mRNA and PrP^C^ in OSCC versus control group from TCGA, GEO and CPTAC databases. (B) Salivary and serum PrP^C^ in OSCC versus OPMD group.

ROC curves were carried out to investigate the diagnostic value of *PRNP* mRNA and PrP^C^ to distinguish OSCC from normal tissues. AUCs of *PRNP* mRNA in TCGA and GEO were 0.852 and 0.94 respectively, with the sensitivity (80.97% and 82.04%) and the specificity (75% and 97.78%). The AUC, sensitivity and specificity of PrP^C^ in CPTAC were 0.812, 71.7% and 95.83% respectively (Fig. 4A).

### The diagnostic value of PrP^C^ in serum and saliva between OSCC and OPMD patients

In accordance to the ROC curve derived from OSCC patients versus control subjects, with the cut-off value of 1.054 ng/ml for salivary PrP^C^, the sensitivity and specificity in distinguishing between OSCC and OPMD patients were 90.79% and 63.33%. The data also revealed a threshold value of 1.222 ng/ml for serum PrP^C^ with the sensitivity of 56.58% and the specificity of 80.00%. In view of the cut-off values, cases exceeding these values would be diagnosed with OSCC. And the AUC for salivary PrP^C^ (0.807, *P* < 0.0001) was a little higher than that for serum PrP^C^ (0.671, *P* < 0.05) (Fig. 4B) (Table 4).

### PrP^C^-related proteins interaction network analysis

**Figure 5:**
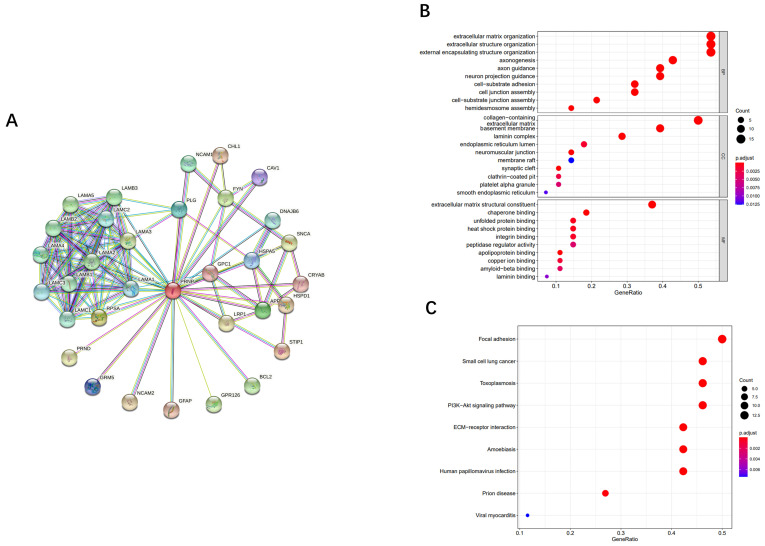
The analysis of proteins interaction network, GO functional enrichment and KEGG pathway enrichment related to PrP^C^. (A) functional PrP^C^-related proteins interaction network constructed from STRING database. (B) GO enrichment analysis of PrP^C^-related DEPs in OSCC from CPTAC database. (C) KEGG enrichment analysis of PrP^C^-related DEPs in OSCC from CPTAC database.

PPI network was conducted through the STRING to predict PrP^C^-related proteins interaction network (Fig. 5A). There were 33 nodes and 119 edges generated with nodes representing proteins and edges representing interactions. The proteins' average node degree was 7.21 and the significant PPI enrichment *p*-value was < 1.0e-16.

### PrP^C^-related DEPs between OSCC and normal tissues from CPTAC

There were a total of 33 proteins interacted with each other, among which 28 proteins were differentially expressed in OSCC compared to normal tissues from CPTAC database. The 28 proteins were PrP^C^-related DEPs between OSCC and control groups. 12 proteins showed up-regulation in the tumor including PrP^C^, GPC1, APP, STIP1, HSPA5, RPSA, LAMA1, LAMA3, LAMB1, LAMB3, LAMC2, DNAJB6 (*P*<0.05). And 16 proteins were down-regulated in the tumor including CAV1, NCAM1, PLG, CHL1, LAMA2, LAMA4, LAMA5, LAMB2, LAMC1, LAMC3, SNCA, CRYAB, HSPD1, LRP1, BCL2, NCAM2 (*P*<0.05). Two proteins (GFAP and FYN) showed no markedly differential expression ((*P*>0.05). And the rest of three proteins (GPR126, PRND and GRM5) have not been found in CPTAC.

### GO functional enrichment and KEGG pathway enrichment analysis

To explore the functions and signaling pathways of PrP^C^ in OSCC*,* we analyzed the twenty-eight PrP^C^-related DEPs of OSCC by GO and KEGG enrichment with the ClusterProfiler package. Biological process (BP), cellular component (CC) and molecular function (MF) are the three ontologies of GO terms. As shown in the bubble chart of Fig. 5B, BP of DEPs has been mainly manifested in extracellular matrix organization, extracellular structure organization and external encapsulating structure organization et al. (*P*<0.05). CC has been significantly enriched in collagen-containing, basement membrane and laminin complex (*P*<0.05). It is also demonstrated that PrP^C^-related DEPs play their molecular functions (MF) through extracellular matrix structural constituent and chaperone binding (*P*<0.05). KEGG pathway analysis was carried out to indicate that PrP^C^ is related to the pathway of prion disease (*P*<0.01), and DEPs are statistically enriched in focal adhesion, small cell lung cancer, toxoplasmosis, PI3K-Akt signaling pathway, ECM-receptor interaction, amoebiasis and human papillomavirus infection (*P*<0.0001) (Fig. 5C).

## Discussion

Identifying carcinogenic biomarkers and elucidating the potential mechanisms for the occurrence and development of OSCC will be of great benefit to the early diagnosis and effective treatment of patients with the highly malignant tumor 29. Up to date, tissue biopsy followed by histological assessment is still considered as the gold standard for distinguishing between OSCC and OPMDs 8, 30. However, early diagnosis of OSCC is often delayed because biopsy is invasive, painful, time-consuming, and requires skilled medical practitioners 31, 32. As a consequence, detecting carcinogenic biomarkers in serum and saliva can be taken advantage as potential detection approaches to guide clinical diagnostic examination 33, 34.

PrP^C^ is a small glycoprotein that is widely anchored to the cell membrane outer leaflet and highly conserved in all mammals 12, 35. It not only exists in nervous system, lymphoid tissues, lung, heart, intestinal tract and other tissues and organs 36, but also is highly expressed in some kinds of tumors, such as gastric cancer, pancreatic cancer, glioblastoma and schwannoma, so as to exert its biological and pathological functions 14, 37. We used the GEPIA website to investigate the expression of *PRNP* mRNA in thirty-three different cancers, and found that it was highly expressed in five kinds of cancers (lymphoid neoplasm diffuse large B-cell lymphoma, esophageal cancer, head and neck squamous cell carcinoma, pancreatic adenocarcinoma and thymoma) compared with adjacent cancer tissues. The result showed that *PRNP* mRNA may become a potential diagnostic biomarker in tumors. And it demonstrated that PrP^C^ may also be overexpressed in OSCC and may play a vital part in this cancer.

In addition, we found that expression of *PRNP* mRNA increased in OSCC compared with paracancerous and normal tissues by TCGA and GEO databases, which was consistent with that mentioned above. We analyzed the CPTAC protein database and proved that the level of PrP^C^ in OSCC tissues was much higher than that in normal tissues. Our previous immunohistochemical experiment also confirmed that it was elevated in OSCC tissues 20. These results suggest that differential content of PrP^C^ may be detected in human serum and saliva, which has been further verified in our ELISA trail. The up-regulation of PrP^C^ in OSCC serum and saliva was the same as the results in the databases and previous experiments. It is concluded that PrP^C^ in serum and saliva may be a useful biomarker for diagnosing OSCC.

Therefore, the diagnostic role of serum and salivary PrP^C^ in OSCC needed to be supplemented and confirmed. The databases showed that *PRNP* mRNA and PrP^C^ had pretty high diagnostic value in recognizing OSCC and control subjects. AUCs were both higher than 0.8, the sensitivity was 80.97%, 82.04% and 71.7%, and the specificity was 75%, 97.78% and 95.83% respectively in TCGA, GEO and CPTAC. What's more, salivary and serum PrP^C^ revealed its early diagnostic value in differentiating OSCC from OPMD patients. The diagnostic value of salivary PrP^C^ (AUC 0.804, sensitivity 90.79% and specificity 63.33%) was better than that of serum (AUC 0.671, sensitivity 56.58% and specificity 80.00%). It was suggested that salivary and serum PrP^C^ had good diagnostic value in distinguishing OSCC from OPMD patients. And salivary PrP^C^ seemed to be a better diagnostic biomarker than serum PrP^C^.

In this study, the relationships between expression of *PRNP* mRNA and PrP^C^ and clinical parameters in OSCC patients were also analyzed. In TCGA, the expression of *PRNP* mRNA was related to age. In CPTAC, the expression of PrP^C^ increased in stage III and IV of OSCC contrasted with stage I and II. To analyze the relationship between salivary and serum PrP^C^ levels and clinicopathological variables in OSCC patients, it was found that moderately and poorly differentiated OSCC patients had higher levels of PrP^C^ in serum and saliva than highly differentiated ones. It has proved that PrP^C^ may be associated with the occurrence and development of OSCC. Ulteriorly, PrP^C^ may interact with other proteins in order to promote the progression of OSCC through possible biological functions and signaling pathways.

It reported that up-regulation of PrP^C^ affected the expression of laminin and integrin α6, thus promoting the cell migration of glioblastoma multiforme. The interaction between PrP^C^ and Notch1 also stimulated the growth and invasion of pancreatic ductal adenocarcinoma. As for melanoma, PrP^C^ modulates actin polymerization through HSP27 and AKT to actively influence cells migrating 38. Interestingly, it has been illustrated that the high level of PrP^C^ was associated with drug resistance of glioblastoma, gastric cancer and breast cancer 14. PrP^C^ can activate the P13K-AKT pathway to increase the expression of MDR1, or interact with CD44 to enhance drug resistance of tumor cells 14, 38. Moreover, a large number of literatures has shown that up-regulated PrP^C^ is related to the prognosis of tumors, such as gastric cancer 39 and glioma 40 as well as head and neck squamous cell carcinoma 41. As a consequence, PrP^C^ is a potential biomarker for early diagnosis, effective treatment and prognosis of tumors.

To understand the probable biological functions of PrP^C^ in OSCC, we first searched for PrP^C^ related interaction proteins on the STRING website. Then the work would be done to find out which of these proteins expressed differentially between OSCC and normal tissues through CPTAC. As a result, 16 proteins were up-regulated while 12 proteins were down-regulated in OSCC. The DEPs in OSCC are related to nervous system, cell division and growth, cell adhesion, cell differentiation, signal transmission, tumor occurrence and development, tumor migration and proliferation, apoptosis, immunity and autophagy 24. Through GO analysis, the DEPs in OSCC mainly contain collagen, and most of them are components of the basement membrane and laminin complex. The biological processes of OSCC may be carried out by extracellular matrix organization, extracellular structure organization and encapsulating structure organization, and molecular function of DEPs may be accomplished by the extracellular matrix structural constituent and chaperone binding. Further exploring the possible signaling pathways of DEPs in OSCC through KEGG, we found that OSCC may share the same biological pathways as small cell lung cancer, toxoplasmosis, amoebiasis and human papillomavirus infection, and the DEPs may participate in pathways of focal adhesion, PI3K-Akt signaling pathway and ECM- receptor interaction. Related research suggests that the pathway of focal adhesion is activated by SEMA3F to induce metastasis of hepatocellular carcinoma leading to poor survival 42, and some focal adhesion proteins have been contributed to the prognosis and therapies of the carcinoma 43. Leupaxin promotes proliferation, angiogenesis and metastasis through the PI3K-Akt signaling pathway, then becomes a potential therapeutic target in bladder cancer 44. Twist2 promotes cell proliferation and is involved in the progression of kidney cancer by the ECM-receptor interaction pathway 45. These discoveries indicate that PrP^C^ may advance the development of OSCC through focal adhesion, PI3K-Akt signaling pathway and ECM- receptor interaction, and therefore, affect the therapeutic effect and prognosis of OSCC patients.

## Conclusion

In summary, salivary and serum PrP^C^ of OSCC patients show increased levels compared with OPMD and control group, and PrP^C^ may be associated with the occurrence and development of OSCC. This study reports salivary and serum PrP^C^ might be used as an important predictive early diagnostic indicator and a possible therapeutic target for OSCC. PrP^C^ may play its pathophysiological functions in OSCC through cell adhesion, cell differentiation, signal transmission and apoptosis.

## Figures and Tables

**Table 1:** Participant information in OSCC, OPMD and control groups

Parameters	OSCC	OPMD	Control
**Total, n**	76	30	78
**Age (year)**			
Range	48-85	42-79	40-78
Mean (SD)	70±3	61±4	60±7
**Gender, n (%)**			
Male	45 (59.2%)	17 (56.7%)	44 (56.4%)
Female	31 (40.8%)	13 (43.3%)	34 (43.6%)
**Lesion sites, n (%)**			
Tongue	24 (31.6%)	12 (40.0%)	--
Maxilla	8 (10.5%)	0 (0%)	--
Mandible	10 (13.2%)	2 (6.7%)	--
Buccal mucosa	12 (15.8%)	11 (36.7%)	--
Hard palate	4 (5.3%)	1 (3.3%)	--
Gingiva	15 (19.7%)	3 (10.0%)	--
Lower lip	3 (3.9%)	1 (3.3%)	--
**Habits, n (%)**			
Ever Smoking	31 (40.8%)	13 (43.3%)	--
Ever Drinking	26 (34.2%)	8 (26.7%)	--
Ever Chewing	14 (18.4%)	2 (6.7%)	--

OSCC oral squamous cell carcinoma, OPMD oral potentially malignant disorder, SD standard deviation

**Table 2:** Salivary and serum PrP^C^ levels in OSCC, OPMD and control groups

Groups	Cases	Salivary PrP^C^ (ng/ml)	*P* value	Serum PrP^C^ (ng/ml)	*P* value
OSCC	76	1.790 ± 0.963	<0.0001^a^	1.251 ± 0.255	0.018^a^
OPMD	30	1.133 ± 0.413	0.516^b^	1.111 ± 0.198	0.523^b^
Control	78	1.229 ± 0.380	<0.0001^c^	1.148 ±0.311	0.020^c^

^a^ Difference between OSCC and OPMD groups ^b^ Difference between OPMD and control groups ^c^ Difference between OSCC and control groups

**Table 3:** Association between salivary and serum PrP^C^ levels and clinicopathological variables in OSCC

Types	Cases	Salivary PrP^C^ (ng/ml)	*P* value	Serum PrP^C^ (ng/ml)	*P* value
**Age (year)**					
<50	22	1.537±0.631	0.208	1.306±0.312	0.739
≥50	54	1.900±1.102		1.267±0.420	
**Gender**					
Male	45	1.879±0.975	0.625	1.308±0.430	0.341
Female	31	1.765±1.123		1.224±0.363	
**Ethnicity**					
Minority	26	2.011±1.252	0.460	1.361±0.325	0.301
Han	50	1.791±0.987		1.250±0.421	
**Differentiation**					
Well	48	1.607±0.723	0.018	1.216±0.381	0.021
Moderate, poor	28	2.381±1.433		1.443±0.428	
**Clinical stages**					
I+II	33	2.050±1.247	0.100	1.336±0.566	0.302
III+IV	43	1.655±0.798		1.231±0.237	
**Lymph node Metastasis**					
Negative	44	1.984±1.147	0.064	1.287±0.487	0.693
Positive	32	1.587±0.780		1.252±0.236	

**Table 4:** Diagnostic value of salivary and serum PrP^C^ in OSCC versus OPMD patients

Biomarker	AUC*	SE	95%CI	Sensitivity (%)	Specificity (%)	PPV (%)	NPV (%)	Diagnostic efficiency (%)	Youden index value	Cut-off point (ng/ml)	*P* value
Salivary PrP^C^	0.807	0.052	0.719 - 0.877	90.79	63.33	86.25	73.08	83.02	0.5412	1.054	<0.0001
Serum PrP^C^	0.671	0.059	0.573 - 0.759	56.58	80.00	87.76	42.11	63.21	0.3658	1.222	0.0035

AUC area under the curve, SE standard error, 95%CI 95% confidence interval, PPV positive predictive value, NPV negative predictive value * Salivary PrP^C^ compared with serum PrP^C^, z =1.772, *P* =0.0764 *P* value, ROC curves compared with reference line

## References

[1] Lu Y, Huang W, Chen H (2019). MicroRNA-224, negatively regulated by c-jun, inhibits growth and epithelial-to-mesenchymal transition phenotype via targeting ADAM17 in oral squamous cell carcinoma. J Cell Mol Med.

[2] Sung H, Ferlay J, Siegel RL (2021). Global Cancer Statistics 2020: GLOBOCAN Estimates of Incidence and Mortality Worldwide for 36 Cancers in 185 Countries. CA Cancer J Clin.

[3] Omar E (2015). Current concepts and future of noninvasive procedures for diagnosing oral squamous cell carcinoma-a systematic review. Head Face Med.

[4] Hsu CW, Chang KP, Huang Y (2019). Proteomic Profiling of Paired Interstitial Fluids Reveals Dysregulated Pathways and Salivary NID1 as a Biomarker of Oral Cavity Squamous Cell Carcinoma. Mol Cell Proteomics.

[5] Park DG, Woo BH, Lee BJ (2019). Serum Levels of Interleukin-6 and Titers of Antibodies Against Porphyromonas gingivalis Could Be Potential Biomarkers for the Diagnosis of Oral Squamous Cell Carcinoma. Int J Mol Sci.

[6] Jain A, Mallupattu SK, Thakur R (2021). Role of Oncofetal Protein CR-1 as a Potential Tumor Marker for Oral Squamous Cell Carcinoma. Indian J Clin Biochem.

[7] Mankapure PK, Barpande SR, Bhavthankar JD (2015). Serum big endothelin-1 as a biomarker in oral squamous cell carcinoma patients: an analytical study. J Appl Oral Sci.

[8] Chakraborty D, Natarajan C, Mukherjee A (2019). Advances in oral cancer detection. Adv Clin Chem.

[9] Melguizo-Rodríguez L, Costela-Ruiz VJ, Manzano-Moreno FJ (2020). Salivary biomarkers and their application in the diagnosis and monitoring of the most common oral pathologies. Int J Mol Sci.

[10] Khurshid Z, Zafar MS, Khan RS (2018). Role of salivary biomarkers in oral cancer detection. Adv Clin Chem.

[11] Patil S, Arakeri G, Alamir AWH (2019). Role of salivary transcriptomics as potential biomarkers in oral cancer: A systematic review. J Oral Pathol Med.

[12] Wiegmans AP, Saunus JM, Ham S (2019). Secreted cellular prion protein binds doxorubicin and correlates with anthracycline resistance in breast cancer. JCI Insight.

[13] Go G, Lee SH (2020). The Cellular Prion Protein: A Promising Therapeutic Target for Cancer. Int J Mol Sci.

[14] Manni G, Lewis V, Senesi M (2020). The cellular prion protein beyond prion diseases. Swiss Med Wkly.

[15] Zhou L, Shang Y, Liu C (2014). Overexpression of PrPc, combined with MGr1-Ag/37LRP, is predictive of poor prognosis in gastric cancer. Int J Cancer.

[16] Li C, Yu S, Nakamura F (2009). Binding of pro-prion to filamin A disrupts cytoskeleton and correlates with poor prognosis in pancreatic cancer. J Clin Invest.

[17] Hinton C, Antony H, Hashimi SM (2013). Significance of prion and prion-like proteins in cancer development, progression and multi-drug resistance. Curr Cancer Drug Targets.

[18] Luo G, Wang W, Wu Q (2017). MGr1-Antigen/37 kDa laminin receptor precursor promotes cellular prion protein induced multi-drug-resistance of gastric cancer. Oncotarget.

[19] Cheng Y, Tao L, Xu J (2014). CD44/cellular prion protein interact in multidrug resistant breast cancer cells and correlate with responses to neoadjuvant chemotherapy in breast cancer patients. Mol Carcinog.

[20] Zhang J, Zeng Y, Zheng J (2013). Expression of Prion protein and its clinical significance in oral squamous cells carcinoma and oral leukoplakia. Zhonghua Kou Qiang Yi Xue Za Zhi.

[21] Zheng J, Sun L, Yuan W (2018). Clinical value of Naa10p and CEA levels in saliva and serum for diagnosis of oral squamous cell carcinoma. J Oral Pathol Med.

[23] Edwards NJ, Oberti M, Thangudu RR (2015). The CPTAC Data Portal: A Resource for Cancer Proteomics Research. J Proteome Res.

[24] Tang Z, Li C, Kang B (2017). GEPIA: a web server for cancer and normal gene expression profiling and interactive analyses. Nucleic Acids Res.

[25] Szklarczyk D, Gable AL, Nastou KC (2021). The STRING database in 2021: customizable protein-protein networks, and functional characterization of user-uploaded gene/measurement sets. Nucleic Acids Res.

[26] Gaudet P, Skunca N, Hu JC (2017). Primer on the Gene Ontology. Methods Mol Biol.

[27] Yu GC, Wang LG, Han YY (2012). ClusterProfiler: an R Package for Comparing Biological Themes Among Gene Clusters. OMICS.

[28] Gaudet P, Škunca N, Hu JC (2017). Primer on the Gene Ontology. Methods Mol Biol.

[29] Yang K, Zhang S, Zhang D (2019). Identification of SERPINE1, PLAU and ACTA1 as biomarkers of head and neck squamous cell carcinoma based on integrated bioinformatics analysis. Int J Clin Oncol.

[30] Yang EC, Tan MT, Schwarz RA (2018). Noninvasive diagnostic adjuncts for the evaluation of potentially premalignant oral epithelial lesions: current limitations and future directions. Oral Surg Oral Med Oral Pathol Oral Radiol.

[31] Neville BW, Day TA (2002). Oral cancer and precancerous lesions. CA Cancer J Clin.

[32] Chattopadhyay I, Panda M (2019). Recent trends of saliva omics biomarkers for the diagnosis and treatment of oral cancer. J Oral Biosci.

[33] Piyarathne NS, Rasnayake RMSGK, Angammana R (2021). Diagnostic salivary biomarkers in oral cancer and oral potentially malignant disorders and their relationships to risk factors - A systematic review. Expert Rev Mol Diagn.

[34] Ni YH, Ding L, Hu QG (2015). Potential biomarkers for oral squamous cell carcinoma: proteomics discovery and clinical validation. Proteomics Clin Appl.

[35] Yang X, Zhang Y, Zhang L (2014). Prion protein and cancers. Acta Biochim Biophys Sin (Shanghai).

[36] Thumdee P, Ponsuksili S, Murani E (2007). Expression of the prion protein gene (PRNP) and cellular prion protein (PrPc) in cattle and sheep fetuses and maternal tissues during pregnancy. Gene Expr.

[37] Mehrpour M, Codogno P (2010). Prion protein: From physiology to cancer biology. Cancer Lett.

[38] Prado MB, Melo Escobar MI, Alves RN (2020). Prion Protein at the Leading Edge: Its Role in Cell Motility. Int J Mol Sci.

[39] Zhou L, Shang Y, Liu C (2014). Overexpression of PrPc, combined with MGr1-Ag/37LRP, is predictive of poor prognosis in gastric cancer. Int J Cancer.

[40] Thellung S, Corsaro A, Bosio AG (2019). Emerging Role of Cellular Prion Protein in the Maintenance and Expansion of Glioma Stem Cells. Cells.

[41] Santos EM, Fraga CAC, Xavier AREO (2021). Prion protein is associated with a worse prognosis of head and neck squamous cell carcinoma. J Oral Pathol Med.

[42] Ye K, Ouyang X, Wang Z (2020). SEMA3F Promotes Liver Hepatocellular Carcinoma Metastasis by Activating Focal Adhesion Pathway. DNA Cell Biol.

[43] Yam JW, Tse EY, Ng IO (2009). Role and significance of focal adhesion proteins in hepatocellular carcinoma. J Gastroenterol Hepatol.

[44] Hou T, Zhou L, Wang L (2018). Leupaxin Promotes Bladder Cancer Proliferation, Metastasis, and Angiogenesis Through the PI3K/AKT Pathway. Cell Physiol Biochem.

[45] Zhang HJ, Tao J, Sheng L (2016). Twist2 promotes kidney cancer cell proliferation and invasion by regulating ITGA6 and CD44 expression in the ECM-receptor interaction pathway. Onco Targets Ther.

